# Psychometric Evaluation of Social Cognitive Measures for Adults with Autism

**DOI:** 10.1002/aur.2084

**Published:** 2019-02-15

**Authors:** Kerrianne E. Morrison, Amy E. Pinkham, Skylar Kelsven, Kelsey Ludwig, David L. Penn, Noah J. Sasson

**Affiliations:** ^1^ School of Behavioral and Brain Sciences The University Of Texas At Dallas Richardson Texas, 75080; ^2^ Department of Psychology and Neuroscience The University of North Carolina at Chapel Hill Chapel Hill North Carolina, 27599; ^3^ School of Psychology Australian Catholic University 3065, Melbourne VIC Australia

**Keywords:** autism spectrum disorder, adults, reliability, social social cognition, validity

## Abstract

Although social cognition is frequently identified as a target in clinical trials and psychosocial interventions for adults with autism spectrum disorder (ASD), these efforts are hampered by a lack of consensus and validation of social cognitive measures. The current study provides psychometric evaluation of 11 frequently used measures encompassing different subdomains of social cognition. Adults with autism (N = 103) and typically developing controls (N = 95) completed 11 commonly used social cognitive tasks spanning the domains of emotion processing, social perception, and mentalizing/theory of mind. We examined each measure's internal reliability and sensitivity to group differences, how performance related to general intellectual ability, and alignment of measures with a proposed two‐factor structure of social cognition in ASD. Controls outperformed the ASD group on 8 of the 11 social cognitive tasks, with the largest group differences occurring on two mentalizing measures, The awareness of social inference task (TASIT) and hinting task. In ASD, all tasks demonstrated strong internal consistency and avoided ceiling and floor effects. Social cognitive performance was also related to, but not redundant with, intellectual functioning. We also found support for a two‐factor structure of social cognition, with basic social perception and emotional processing aligning into a lower‐order social perception factor, while mentalizing tasks aligned into a higher‐order social appraisal factor. In sum, eight tasks showed adequate to strong psychometric properties. The psychometric data, effect size estimates, and correlations between measures reported here can be used for study planning for social cognitive interventions in autism. ***Autism Research** 2019, 12: 766–778*. © 2019 The Authors. *Autism Research published by International Society for Autism Research* published by Wiley Periodicals, Inc.

**Lay Summary:**

We examined 11 tasks that measure how adults with autism perceive and interpret social information. Eight of the tasks were reliable and showed lower performance in adults with autism compared to typically‐developing controls. Task performance was related to but distinguishable from IQ. These measures evaluated here may be useful in assessing the effectiveness of interventions and treatments to improve social abilities in adults with autism.

## Introduction

Social cognition refers to the ability to perceive and interpret social information [Brothers, 1990] and is broadly impaired in disorders characterized by social impairments such as schizophrenia and autism spectrum disorder (ASD), including in those without intellectual disability [Baron‐Cohen, Jolliffe, Mortimore, & Robertson, 1997; Heavey, Phillips, Baron‐Cohen, & Rutter, 2000; Klin, 2000; Klin, Jones, Schultz, Volkmar, & Cohen, 2002; Sasson, Pinkham, Carpenter, & Belger, 2011; Klin et al., 2002]. This has prompted an examination of social cognitive abilities in both populations, with particular interest in how individuals with these clinical conditions perform on social cognitive tasks relative to controls [Pinkham, Penn, Green, & Harvey, 2015; Klin et al., 1999; Sasson et al., 2007]. In schizophrenia, poor social cognitive ability predicts social skills and daily living skill ability, suggesting social cognition is an important area to target in treatments [Pinkham, Harvey, & Penn, 2017]. Social cognitive challenges may also relate to difficulties with social functioning for adults with ASD [Sasson et al., 2011], but training programs developed to target social cognition in ASD have shown inconsistent effects [Bishop‐Fitzpatrick, Minshew, & Eack, 2014] and often result in only modest improvements that do not generalize well to real‐world functioning [Gates, Kang, & Lerner, 2017]. The inconsistency and limited efficacy of these programs may occur in part because of the variable ways social cognition is operationalized and measured across treatment and research studies with ASD adults [Kliemann & Adolphs, 2018].

Until relatively recently, social cognitive research and treatment in ASD largely focused on extending validated methodologies developed for children to adult populations, which has resulted in tasks that are inadequately sensitive and limited in their effect [Baron‐Cohen et al., 1997; Roeyers, Buysse, Ponnet, & Pichal, 2001]. Examined independently, these studies commonly—but not uniformly—indicate that individuals with ASD remain impaired in adulthood across many domains of social cognition relative to typically‐developing (TD) controls, including the detection of emotional biological motion [Hubert et al., 2007], social orienting [Sasson et al., 2007], face scanning [Pelphrey et al., 2002], face recognition [Klin et al., 1999; Sasson, 2006], affect recognition [Eack, Mazefsky, & Minshew, 2015; Loveland et al., 1997], and advanced theory of mind [Baron‐Cohen et al., 1997]. However, sample characteristics and tasks differ between studies, making it hard to evaluate patterns of social cognitive ability in ASD across domains and to isolate areas of relative strength and weakness. An analysis of the factor structure of social cognition in ASD adults, and a psychometric evaluation of commonly used tasks of social cognition in ASD, can help assess the relevance of social cognition for this population and provide recommendations for the best tools to evaluate its subdomains in ASD research, treatment, and clinical trials [National Institute of Mental Health, 2016].

Fortunately, a model exists to facilitate this process. In research on schizophrenia, a clinically distinct condition from ASD that also is characterized by social dysfunction in adulthood, a need emerged to systematically evaluate the psychometric properties of social cognitive measures to make recommendations for use in clinical trials [Carter & Barch, 2007; Gold, 2012; Kern et al., 2013; Pinkham et al., 2013]. The social cognition psychometric evaluation (SCOPE) study [Pinkham et al., 2013] was developed to identify core domains of social cognition, select measures assessing each domain, and empirically test the reliability and validity of each measure. To do this, surveys of schizophrenia and ASD experts were used in conjunction with the RAND panel approach to identify the core domains of social cognition and achieve a consensus on the best tasks to assess and represent those domains [Fitch, Bernstein, Aguilar, Burnand, & LaCalle, 2001; Kern et al., 2013; Pinkham et al., 2013]. The nominated tasks were then tested for their psychometric properties [Pinkham et al., 2015].

Four core domains of social cognition were identified: emotion processing (e.g., ability to recognize emotional expressions from faces and vocal tone), social perception (e.g., understanding social roles and rules, and interpreting social cues), mental state attribution/theory of mind (e.g., inferring the mental states, intentions, thoughts, and emotions of others), and attributional style/bias [e.g., tendencies in the way one explains social phenomena and situations; Pinkham et al., 2015]. Eight tasks bridging the four domains were selected by expert consensus for psychometric evaluation (see in Table 1) with several tasks of emotion processing and theory of mind showing strong psychometric properties [Pinkham et al., 2015]. Other nominated tasks, such as the measure of attributional style [ambiguous intentions and hostility questionnaire; Combs, Penn, Wicher, & Waldheter, 2007] and the trustworthiness task [Adolphs, Tranel, & Damasio, 1998] performed poorly and were not recommended for further use. Moreover, the single social perception measure [relationships across domains task; Sergi et al., 2009] showed weak psychometric properties, suggesting this domain may not yet be measured adequately in schizophrenia.

**Table 1:** Social Cognitive Task Recommendations by the Social Cognition Psychometric Evaluation [Pinkham et al., 2013]

Domain	Task	Description	Citation	SCOPE Recommendation
Emotion processing	Penn emotion recognition task (ER40)	Facial recognition task of static faces	Kohler et al., 2003	Adequate
	Bell Lysaker emotion recognition task (BLERT)	Facial recognition task of dynamic faces	Bryson et al., 1997	Strong
Social Perception	Relationships across domains (RAD)	Identification of different social relationships using vignettes	Sergi et al., 2009	Weak
Theory of Mind	The awareness of social inference task (TASIT)	Identification of thoughts, feelings, and intentions of characters in video vignettes	MacDonald et al., 2003	Adequate
	Reading the mind in the eyes (Eyes)	Recognition of emotion and cognitive states of static faces	Baron‐Cohen et al., 2001	Adequate
	Hinting task (Hinting)	Identification of vignette characters' thoughts and feelings	Corcoran et al., 1995	Strong
Attribution Style/Bias	Ambiguous intentions and hostility questionnaire (AIHQ)	Measure of attributing hostility and feelings of blame towards vignette characters	Combs et al., 2007	Weak
Additional Measure	Trustworthiness (Trust)	Rating of static faces on how trustworthy they appear	Adolphs et al., 1998	Weak

In studies directly comparing adults with ASD and those with schizophrenia, not only do both groups demonstrate poorer performance in social skills [Morrison et al., 2017], social functioning [APA, 2013], and social cognitive performance [Sasson et al., 2011] relative to TD controls, but they also show similar underactivation in brain regions associated with social cognition [Couture et al., 2010; Crespi, Stead, & Elliot, 2010; Pinkham, Hopfinger, Pelphrey, Piven, & Penn, 2008; Sullivan et al., 2012]. Other studies, however, show distinctions in aspects of social cognition, such as greater impairments in social perception in ASD [e.g., face processing; Pilowsky, Yirmiya, Arbelle, & Mozes, 2000; Sasson et al., 2007; Sasson, Pinkham, Weittenhiller, Faso, & Simpson, 2016] but fewer impairments in higher‐order social appraisal [e.g., theory of mind and attributional biases Pinkham et al., 2012; Sasson et al., 2011]. These differences suggest that social cognitive performance and domains may be related in different ways for ASD and schizophrenia. Whereas social cognitive performance aligns with a one‐factor structure in schizophrenia [Browne et al., 2016], recent work suggests a two‐factor structure in ASD, in which a lower‐order social perception ability is distinct from, but a prerequisite to, social appraisal processes [Sasson et al., 2011]. These differences also suggest that the psychometric properties established for social cognitive tasks using a schizophrenia sample may not be applicable to ASD adults. Therefore, an independent assessment is needed to examine social cognitive performance specifically in ASD.

The current study aimed to psychometrically evaluate social cognitive tasks for adults with ASD and TD controls within a large sample. Measures selected and recommended by SCOPE [Pinkham et al., 2013; Pinkham et al., 2015] were examined here, including measures of emotion processing, mentalizing, and social perception. The AIHQ task was not included here because, unlike in schizophrenia, hostile attributions are not considered a social cognitive bias characteristic of autism. Instead, several tasks relevant to autism were added to assess face processing [Benton facial recognition task; Benton & Van Allen, 1968], biological motion detection [emotional biological motion task and basic biological motion; Heberlein, Adolphs, Tranel, & Damasio, 2004; Puce & Perrett, 2003], and theory of mind [Cartoon Theory of Mind; Brüne, 2003].

Reliability, utility, and validity were examined for each task. We expected emotion processing and mentalizing tasks to demonstrate adequate to strong reliability and limited floor and ceiling effects. We next examined whether the tasks aligned into two primary domains of social cognition. We predicted one factor would be composed of social perception tasks such as recognition of faces and emotion processing, while higher‐order social cognitive abilities such as mentalizing/theory of mind would comprise the second factor. Factors were then used to interpret patterns of results in evaluations of validity. To assess validity, we first examined each task's ability to differentiate between ASD and TD groups. We then examined associations between tasks, and the relationships between task performance and IQ. Here, we predicted moderate to strong correlations with IQ, but we predicted IQ would be more strongly related to the higher‐order social appraisal tasks compared to the lower‐order emotion processing tasks due to their greater cognitive demands. We also predicted that social perception and social appraisal tasks would be more strongly related to each other compared to each domain's relationship with IQ.

## Methods

### *Participants*

Participants with ASD (*n* = 103) were recruited from the nonPareil Institute, a local nonprofit organization, and from the local and university community. Diagnoses were confirmed using the autism diagnostic observation schedule [ADOS‐2; Lord et al., 2012]. TD adults (*n* = 95) were recruited from the local community in Dallas, Texas and Chapel Hill, North Carolina for a multisite study assessing social cognition and functioning in adults with schizophrenia. The TD participants recruited from each site did not differ on performance on the social cognitive tasks (*Ps* > .07). Because TD data were collected for a larger project, TD participants were selected from the larger dataset (*n* = 146) to match to the ASD population in gender and ethnicity, age, and verbal IQ (described below). Sample characteristics are displayed in Table 2. The institutional review boards at UT Dallas and the University of North Carolina at Chapel Hill reviewed and approved the study, and all participants provided written informed consent and were compensated for participation.

**Table 2:** Sample Demographic Characteristics

	ASD (*n* = 103)				TD (*n* = 95)			
	*n*	%			*n*	%	*Χ* ^*2*^ e	*P*
Male	92	89.3%			84	88.4%	0.04	0.841
Race							0.51	0.774
Caucasian	91	88.3%			86	90.5%		
African American	4	3.9%			4	4.2%		
Asian	8	7.8%			5	5.3%		
	Range	*M*	*SD*		Range	*M*	*SD*	*F*
Age	18–55	24.28	6.17		18–59	24.17	6.21	0.02
WRAT‐3^a^	49‐123	105.64	12.81		77–121	107.64	10.01	1.48
WASI Full‐Scale IQ^b^^,^ c	75–132	108.90	14.46		84–135	116.28	9.82	10.01^d^
WASI Verbal IQ T Score^b^^,^ c	22–76	53.70	12.29		40–72	59.89	7.22	10.26^d^
WASI Nonverbal IQ T Score^b^^,^ c	33–70	56.09	7.76		40–67	58.53	6.15	3.60

a WRAT‐3 = Wide range achievement test—third edition.

b WASI = Wechsler abbreviated scale for intelligence.

c WASI scores were available from a subsample of the data (ASD *n* = 101; TD *n* = 47).

d *P* < 0.05.

e Chi square degrees of freedom for gender was 1, and 2 for race.

### *Procedure*

All but two participants with ASD (*n* = 101) had completed the Wechsler abbreviated scale for intelligence [WASI; Wechsler, 2008] in a prior study session, and a subset of TD participants (*n* = 47) completed the WASI as the first task before being administered the social cognitive measures. All participants completed a proxy for verbal IQ [i.e., the wide range achievement test; WRAT‐3; Wilkinson, 1993], followed by a battery of tasks assessing the domains of social cognition and social skills [findings concerning social skills can be found in Morrison et al., 2017]. The order administering domains (e.g., social cognition, social skills) and tasks within each domain were both counterbalanced. There were no time limits on any of the tasks.

### *Measures*

Emotion Processing. Three tasks measured emotion processing: the Bell Lysaker emotion recognition task [BLERT; Bryson, Bell, & Lysaker, 1997], the Penn emotion recognition task [ER‐40; Kohler et al., 2003], and the emotional biological motion task [Heberlein et al., 2004; Kern et al., 2013]. All stimuli in tasks were shown to participants on a computer, and participants verbally selected answers that were then recorded by a research assistant using pencil and paper. The *BLERT* displays 21 ten‐second video clips of an actor dynamically expressing one of seven emotional states: happiness, sadness, fear, disgust, surprise, anger, or no emotion. After the video, participants select which emotion was expressed. The BLERT was scored for total correct out of 21.

The *ER‐40* presents 40 color photos of static faces depicting one of five emotional states: happiness, sadness, anger, fear, or neutral emotion. Faces are counterbalanced for gender, age, and ethnicity of the face as well as intensity of the emotion. The ER‐40 was scored for correct responses out of 40.

*Emotional Biological Motion* assesses participants' ability to detect emotion in biological motion using 5–10 s videos of 24 point‐light walkers. After the video, participants select one of five emotional states that best characterize the movement: fear, anger, happiness, sadness, or neutral. This task is scored relative to the answers given by the TD reference group [Heberlein et al., 2004]. For each video, answer choices were assigned a proportional value based on the distribution of emotions for that item in the TD sample. Thus, higher values on an item indicated that a higher proportion of the TD adults chose that emotion as the answer. The total correctness score on this task averaged the proportion scores on all items.

Social Perception. Three tasks examined social perceptions: the relationships across domains task [RAD; Sergi et al., 2009], basic human biological motion [Kern et al., 2013, 40], and Benton facial recognition task [Benton & Van Allen, 1968]. The *RAD* measures understanding of different types of social relationships. Participants read 15 vignettes depicting male–female pairs and answer three yes/no questions requiring an understanding of how the pair would act in other situations. The RAD was scored for correct responses out of 45. All vignettes were read aloud to participants and research assistants recorded participant answers.

The *Basic Human Biological Motion* task measures the ability to detect human biological motion using brief videos of point‐light displays. Participants completed this task on a computer that recorded responses. Participants view two blocks of dots either moving randomly or coherently (i.e., a human movement such as walking). After viewing the video, participants then rate whether the motion displayed was a human in motion or dots moving randomly. In block one, dots move in either 100% coherent human motion or 100% random motion. In block two, coherency is manipulated in three conditions: 0% coherent, 70% coherent, and 85% coherent. Videos are presented randomly with 40 trials for each type of coherency. Sensitivity to detect human motion is computed using d‐prime for each level of difficulty (100, 85, and 70%). However, because the three d‐primes exhibited high multicolinearity, the indices were averaged to obtain a biological motion average score.

The *Benton* assesses participants' ability to recognize non‐emotional faces presented in a book of facial stimuli. Participants are shown one face and must choose a matching face from an array of six faces. This task is scored for total correct out of 54, and the administering researcher records each response.

Mentalizing/Theory of Mind. Four tasks assessed mentalizing: the awareness of social inference task [TASIT; McDonald, Flanagan, Rollins, & Kinch, 2003], hinting task [Corcoran, Mercer, & Frith, 1995], reading the mind in the eyes task [Baron‐Cohen, Wheelwright, Hill, Raste, & Plumb, 2001], and the cartoon theory of mind intentions subscale [CToM Intentions; Brüne, 2003]. The TASIT, Eyes, and CToM were shown to participants on a computer and a researcher recorded participants' verbal responses. The hinting Task was read aloud and scored by a researcher. *TASIT* measures the ability to infer others' intentions, thoughts, and feelings. Participants watch 16 vignettes depicting characters either lying or using sarcasm. After each scene participants answer yes/no questions about what the characters are doing, thinking, saying, and feeling. This task is scored for total correct out of 64.

The *Hinting* task measures ability to infer others' true intentions from indirect speech. Participants read 10 vignettes of two characters interacting, ending with one character hinting at his or her true thoughts, feelings, or intentions. Participants give open‐ended responses of what the character truly meant, and if their answer is wrong, the experimenter reads a second hint. Answering correctly on the first hint yields two points, while the second hint yields one. The hinting task is scored for total correct out of 20.

The *Reading the Mind in the Eyes* task measures participants' ability to infer mental states from viewing only the eyes of a face. Participants view 36 black and white static photos and choose one of four descriptors that best represents the mental state expressed in the photo. This task is scored for total correct out of 36.

The *CToM* measures nonverbal mental state attribution. Participants are shown a series of three cartoon panels depicting a character doing something (e.g., a man chopping wood). Participants then select from three choices what happens next (e.g., the man using the wood to build a fire in a fireplace). The 14 item intention subscale measures mental states attribution.

Additional Measure. As recommended from the SCOPE study [Pinkham et al., 2015], the *Trustworthiness* task [Adolphs et al., 1998] was added to the social cognitive battery because it conceptually aligns with multiple social cognitive domains. This task measures the ability to make a complex social judgment of trustworthiness from a series of 42 static black and white faces of varying age, gender, and race displayed to a participant on a computer screen. Researchers recorded participants' verbal ratings of how much they would trust that person using a Likert scale ranging from −3 (not at all trustworthy) to +3 (very trustworthy). The average rating across all faces was used as the outcome variable.

Intelligence Quotient. Participants completed at least one of two tests of general cognitive ability administered by a research assistant. All participants completed the wide range achievement test [WRAT‐3; Wilkinson, 1993], in which participants read 42 words aloud, and a score based on correct pronunciation was converted to a standardized verbal score that approximated intelligence quotient (IQ) [Johnstone, Callahan, Kapila, & Bouman, 1996]. To provide a more comprehensive assessment of cognitive ability, the WASI was completed by a subset of the sample. Participants first completed a verbal section, defining up to 42 words that were scored by a trained research assistant using a manual. Participants then completed 35 matrix reasoning questions, picking one of five choices to complete a pattern. Raw scores were converted to t‐scores for each section, and these scores were used to calculate the standardized IQ. The WRAT‐3 was used to match groups on approximated IQ because WASI data were not available for all participants. The full‐scale WASI and verbal and nonverbal t‐scores were used in analyses because they represent more robust measures of intellectual functioning.

### *Data Analytic Plan*

Distributions of scores on each social cognitive task were generally normal, with skew for each within acceptable limit, and all missing values were deleted list wise. First, reliability was assessed with Cronbach's alpha, where tasks with strong internal consistency yielded an alpha greater than 70 [Peterson, 1994]. Second, we tested for ceiling and floor effects by computing each participants' percent correct on each task and comparing the average correctness to either chance (e.g., 50% correct) or ceiling (e.g., 100% correct) using a one sample *t*‐tests. For basic human biological motion, the hit rate was compared to ceiling (i.e., 1.00), and floor effects (i.e., 0.50), and these effects were not tested for the trustworthiness or emotional biological motion tasks as these have no objective correct answers.

Validity was assessed in a number of ways. First, we examined the factor structure of the social cognitive tasks using exploratory factor analyses (EFA) estimating effects with maximum likelihood estimation and Promax rotation due to the non‐orthogonality of the tasks. We predicted a priori the measures would align into two factors: social perception (e.g., emotion processing and social perception tasks) and social appraisal [e.g., theory of mind and mental state attribution tasks; Sasson et al., 2011]. However, because some tasks may include both perception abilities and higher‐order appraisal (e.g., the mind in the eyes), we sought to explore how the tasks aligned without placing constraints. Second, we examined sensitivity to group differences for each measure by comparing the TD and ASD groups' performance on tasks with a MANOVA. The TD group was predicted to outperform the ASD group on all measures, with effect sizes ranging from small to medium and being larger for social appraisal relative to social perception tasks. Third, we examined the tasks' relationships to each other by computing Pearson's *r* correlation coefficients. Correlations were expected to be larger for tasks within each of the two predicted social cognitive factors than between them. Lastly, to assess the validity of social cognition above and beyond general cognition, we examined the relationship between performance on social cognitive tasks with WASI full‐scale IQ, verbal IQ, and nonverbal IQ. Performance on social cognitive tasks was predicted to correlate significantly but not entirely with IQ, and vary as a function of the cognitive demands of the task, with correlations being stronger for social appraisal tasks than social perception tasks.

## Results

### *Reliability and Ceiling/Floor Effects*

Cronbach's alpha for each task is displayed in Table 3. For the ASD group, tasks exhibited adequate to strong internal consistency, with the alpha values ranging from 0.67 to 0.92. However, for TD adults, internal consistency was only strong for the TASIT, CToM, trust, and biological motion tasks, with the other tasks (i.e., Benton, RAD, mind in the eyes, trustworthiness, BLERT, ER40, Emotional Biological Motion) showing lower levels of internal reliability, especially for the hinting task (α = 0.40). There was no evidence for ceiling or floor effects (*Ps* < .01) and both groups scored between 67 and 87% correct on tasks. Accuracy and the percentage of participants scoring at floor and ceiling are displayed in Table 4.

**Table 3:** Internal Consistency of Social Cognitive Tasks

	ASD	TD
Social cognitive task	Cronbach's α	Cronbach's α
Benton	0.72	0.61
Biological motion	0.90	0.85
BLERT^a^	0.72	0.44
CToM intentions^b^	0.72	0.75
Emotional biological motion	0.79	0.41
ER40^c^	0.67	0.47
Eyes	0.73	0.61
Hinting	0.74	0.40
RAD^d^	0.81	0.63
TASIT^e^	0.86	0.77
Trustworthiness	0.92	0.90

a BLERT = Bell Lysaker emotion recognition.

b CToM = Cartoon theory of mind task.

c ER‐40 = Penn emotion recognition task.

d RAD = Relationships across domains task.

e TASIT = The awareness of social inference task.

**Table 4:** Group Performance and Differences on Social Cognitive Tasks

		TD						ASD							
		*M*	*SD*	% Correct	% Floor^a^	% Ceiling^i^		*M*	*SD*	% Correct	% Floor^a^	% Ceiling^i^		*F* (1, 175)	*d* b
Social Perception	Benton	46.29	3.56	85.77	0	0		43.38	4.80	80.29	1.0	0		20.48^c^	0.49
	Bio Motion	2.55	0.83	75.50	4.9	0		2.50	0.73	77.14	3.1	0		0.14	0.04
	BLERT^d^	17.74	2.02	84.06	0	4.2		16.74	3.14	79.43	4.9	2.9		6.21^c^	0.27
	CToM^e^	11.46	2.58	79.53	10.1	15.7		11.80	2.26	83.98	11.7	22.3		0.86	−0.10
	Emo Bio Motion	33.04	2.61	–	–	–		30.74	4.54	–	–	–		16.29^c^	0.44
	ER‐40^f^	33.65	2.67	84.02	0	0		32.04	3.81	80.19	1.0	1.0		10.21^c^	0.34
Social Appraisal	Eyes	26.74	3.98	71.73	4.2	0		24.19	5.10	67.28	16.2	0		13.50^c^	0.40
	Hinting	17.10	1.68	86.16	0	3.2		14.63	3.62	73.45	10.7	1.9		32.05^c^	0.62
	RAD^g^	34.00	4.23	72.69	2.1	0		30.81	6.45	68.67	15.5	0		14.62^c^	0.41
	TASIT^h^	55.49	5.03	86.68	0	0		48.77	8.11	76.50	1	0		42.24^c^	0.70
	Trustworthiness	0.15	0.54	–	–	–		0.02	0.69	–	–	–		1.99	0.15

a % Floor refers to percentage of each sample scoring at or below 50% on each task.

b Cohen's *d* calculated with the pooled standard deviation as the standardizer.

c *P* < 0.01%.

d BLERT = Bell Lysaker emotion recognition.

e CToM = Cartoon theory of mind task.

f ER‐40 = Penn emotion recognition task.

g RAD = Relationships across domains task.

h TASIT = The awareness of social inference task.

i % Ceiling refers to the percentage of each sample with a perfect score.

### *Factor Structure of Social Cognitive Tasks*

Next, we examined whether the factor structure of the social cognitive tasks aligned into the two predicted factors of social perception and social appraisal [Sasson et al., 2011]. For the ASD sample, two factors were supported with eigenvalues greater than one, explaining 59.67% of the variance and adequate goodness of fit (*Χ* ^*2*^[34] = 42.58, *P* = .15). The pattern matrices of factor loadings are displayed in Table 5. Consistent with hypotheses, tasks measuring social perception loaded onto factor one, and tasks measuring social appraisal loaded onto factor two. The first factor included tasks measuring emotion processing (i.e., ER‐40, BLERT, and Emotional Biological Motion) and social perception (i.e., Biological Motion, Benton). Additionally, mind in the eyes and CToM, which have been conceptualized and promoted as social appraisal tasks, loaded onto the social perception factor, suggesting inconsistency with the other social appraisal tasks in adults with ASD. The second factor, social appraisal, was characterized by tasks related to theory of mind (e.g., TASIT, hinting), as well as the trustworthiness task and the RAD. Although the RAD has been characterized as a social perception measure, the factor loadings suggested this task aligns more with social appraisal and mental state attribution tasks in ASD.

**Table 5:** Exploratory Factor Analysis Pattern Loadings for Social Cognitive Factors^a^

	ASD		TD		
Task	Social perception factor^b^	Social appraisal factor^b^	Social appraisal factor^b^	Face processing factor^b^	Higher order appraisal factor^b^
Benton	**0.777**	−0.177	−0.067	**1.033**	−0.031
Bio Motion^c^	**0.753**	−0.121	**0.484**	0.058	0.116
BLERT^d^	**0.633**	0.204	**0.609**	−0.045	−0.09
CToM^e^	**0.487**	0.262	**0.771**	0.053	0.013
EmoBio Motion^f^	**0.751**	−0.073	**0.233**	0.173	0.145
ER‐40^g^	**0.688**	0.01	0.226	**0.348**	−0.038
Eyes	**0.513**	0.353	**0.789**	0.062	−0.227
Hinting	−0.324	**1.022**	0.053	−0.128	**0.388**
RAD^h^	0.212	**0.636**	**0.475**	−0.107	0.133
TASIT^i^	0.321	**0.574**	**0.45**	0.028	0.403
Trust	0.017	**−0.262**	−0.159	0.066	**0.687**

a Maximum likelihood estimation with Promax rotation.

b Coefficients shown in bold load onto the factor.

c Bio Motion = Biological motion task.

d BLERT = Bell Lysaker emotion recognition.

e CToM = Cartoon theory of mind task.

f Emo Bio Motion = Emotional biological motion task.

g ER‐40 = Penn emotion recognition task.

h RAD = Relationships across domains task.

i TASIT = The awareness of social inference task.

The EFA results suggested three factors were present with eigenvalues greater than one for TD adults. Thus, the EFA was re‐run with a three‐factor solution to fit the data, explaining 55.03% of the variance and showing adequate goodness of fit (*Χ* ^*2*^[25] = 26.36, *P* = .39). The Benton and ER‐40 loaded onto one factor, suggesting this factor represents ability to process information from static faces. Measures of theory of mind most strongly loaded onto another factor (e.g., mind in the eyes, CToM), along with the BLERT and biological motion, indicating this factor represents higher‐order mental state attributions and attributions to dynamic stimuli. The final factor was characterized by the hinting task and trustworthiness task, suggesting the final factor is characterized by higher‐order judgments.

### *Group Differences*

Group means and standard deviations on each task are displayed in Table 4. The overall MANOVA for group differences was significant (λ = 0.01, *F*(11, 165) = 2797.70, *P* < .001, partial η^2^ = 0.995) and one‐way ANOVA follow up tests indicated the TD group significantly outperformed the ASD group on every task except trustworthiness, biological motion, and the CToM, (*Ps* > .16). As predicted, group differences were large on social appraisal tasks, particularly the TASIT and Hinting Task (Cohen's *ds* > 0.62) and smaller for social perception tasks (Cohen's *ds* = 0.34–0.49). Results of ANOVAs and Cohen's *ds* are displayed in Table 4.

### *Relationship between Social Cognitive Tasks*

Correlations between social cognitive tasks are displayed in Table 6. For ASD adults, the social appraisal tasks were strongly correlated to one another (*rs* = 0.46–0.67) with the exception of the trustworthiness task which demonstrated small negative correlations with other social appraisal (*rs* < −0.23) and social perception tasks (*rs* < −0.18). The social perception tasks were moderately to strongly related (*rs* = 0.26–0.61). The relationships between social perception and social appraisal tasks were small to large, with the hinting task showing the weakest correlations with social perception measures (*rs* = 0.13–0.42) and the BLERT showing the strongest (*rs* = 0.39–0.65). For TD adults, social cognitive tasks were minimally to moderately correlated (*rs* = −0.02–0.54), with the strongest relationship between the CToM and TASIT (*r* = 0.54), and weakest between the hinting task (*rs* = −0.02–0.21) and trustworthiness tasks (*rs* = 0.03–0.36) with the other measures.

**Table 6:** Zero‐Order Correlations between Social Cognitive Tasks. ASD correlations below the diagonal, TD above.

		Benton	Bio Motion^c^	BLERT^d^	CToM^e^	Emo Bio Motion^f^	ER‐40^g^	Eyes	Hinting	RAD^h^	TASIT^i^	Trust
Social Perception	Benton	1	.257^a^	0.126	.337^b^	.268^a^	.343^b^	.289^b^	−0.019	0.056	.256^a^	0.165
	Bio Motion^c^	.380^b^	1	.329^b^	.508^b^	.305^b^	.272^a^	.357^b^	0.207	0.105	.342^b^	0.164
	BLERT^d^	.539^b^	.485^b^	1	.445^b^	0.203	.224^a^	.353^b^	0.01	.266^b^	.245^a^	0.122
	CToM^e^	.278^b^	.528^b^	.454^b^	1	.239^a^	.229^a^	.535^b^	0.146	.319^b^	.525^b^	0.135
	Emo Bio Motion^f^	.344^b^	.570^b^	.380^b^	.505^b^	1	.299^b^	.245^a^	0.011	.357^b^	.288^b^	0.169
	ER‐40^g^	.504^b^	.423^b^	.609^b^	.406^b^	.256^b^	1	.273^b^	0.062	.219^a^	.227^a^	0.034
Social Appraisal	Eyes	.518^b^	.459^b^	.611^b^	.573^b^	.402^b^	.469^b^	1	0.097	.402^b^	.371^b^	0.058
	Hinting	0.128	.217^a^	.392^b^	.420^b^	0.125	.260^b^	.463^b^	1	.269^b^	.250^a^	.362^b^
	RAD^h^	.383^b^	.336^b^	.557^b^	.463^b^	.323^b^	.455^b^	.627^b^	.580^b^	1	.434^b^	0.167
	TASIT^i^	.354^b^	.446^b^	.653^b^	.464^b^	.331^b^	.500^b^	.643^b^	.559^b^	.672^b^	1	.331^b^
	Trust	−0.072	−0.115	−0.176	−0.155	−0.007	−0.156	−0.15	‐.217^a^	−0.161	‐.229^a^	1

a *P* < 0.05.

b *P* < 0.01.

c Bio Motion = Biological motion task.

d BLERT = Bell Lysaker emotion recognition.

e CToM = Cartoon theory of mind task.

f Emo Bio Motion = Emotional biological motion task.

g ER‐40 = Penn emotion recognition task.

h RAD = Relationships across domains task.

i TASIT = The awareness of social inference task.

### *Associations between Social Cognition and IQ*

Correlations between IQ and social cognitive task performance are displayed in Table 7. For ASD adults, IQ was most strongly related to social appraisal tasks (e.g., TASIT, RAD) as well as social perception tasks with high language demands (e.g., mind in the eyes, BLERT), and was not significantly related to performance on the trustworthiness task. Verbal IQ was also strongly associated with performance on all tasks except the trustworthiness task. Nonverbal IQ was moderately to strongly related to social cognitive performance on all measures, except the hinting task. For TD adults, full scale IQ was moderately to strongly related to performance on the CToM, RAD, and mind in the eyes tasks but not related to performance on the hinting task, ER‐40, or trustworthiness task. Verbal IQ was also moderately to strongly related to performance on the hinting task.

**Table 7:** Correlations between Social Cognitive Tasks and Measures of IQ

		ASD			TD		
		WASI^j^ full scale IQ (*n* = 101)	WASI^j^ verbal IQ (*n* = 101)	WASI^j^ nonverbal IQ (*n* = 101)	WASI^j^ full scale IQ (*n* = 47)	WASI^j^ verbal IQ (*n* = 47)	WASI^j^ nonverbal IQ (*n* = 47)
Social Perception	Benton	.341^a^	.210^b^	.416^a^	0.077	−0.039	0.193
	Bio Motion^c^	.436^a^	.268^a^	.554^a^	0.049	−0.047	0.147
	BLERT^d^	.524^a^	.422^a^	.496^a^	0.149	0.286	−0.06
	CToM^e^	.605^a^	.475^a^	.608^a^	.513b	.420^a^	.439^a^
	Emo Bio Motion^f^	.381^a^	.244^b^	.468^a^	.290^b^	0.250	0.216
	ER‐40^g^	.452^a^	.340^a^	.490^a^	−0.084	−0.113	0.007
	Eyes	.695^a^	.635^a^	.515^a^	.404^a^	.322^b^	.348^b^
Social Appraisal	Hinting	.565^a^	.655^a^	.213^b^	.314^b^	.363^b^	0.152
	RAD^h^	.786^a^	.745^a^	.532^a^	.533^a^	.522^a^	.348^b^
	TASIT^i^	.637^a^	.654^a^	.368^a^	0.278	0.244	0.218
	Trustworthiness	−0.161	−0.184	−0.097	0.057	−0.018	0.134

a *P* < 0.01.

b *P* < 0.05.

c Bio Motion = Biological motion task.

d BLERT = Bell Lysaker emotion recognition.

e CToM = Cartoon theory of mind task.

f Emo Bio Motion = Emotional biological motion task.

g ER‐40 = Penn emotion recognition task.

h RAD = Relationships across domains task.

i TASIT = The Awareness of social inference task.

j WASI = Wechsler abbreviated scale of intelligence.

## Discussion

Social cognition is a widely studied construct in autism research, yet research and treatment studies focused on social cognition in ASD have been limited by a lack of well‐validated tasks with established psychometric data. Inadequate measurement threatens the validity and replicability of findings, impairs comparability between studies, and can lead to faulty conclusions in clinical trials. The current study sought to address these challenges by comprehensively evaluating the psychometric properties of 11 social cognitive tasks spanning three domains (emotion processing, social perception, and mentalizing) within a sample of 103 adults with ASD and 95 TD controls.

Eight tasks (Benton, BLERT, emotional motion, ER‐40, Eyes, hinting, RAD, and TASIT) demonstrated acceptable psychometric properties. Each displayed strong internal reliability, were not affected by ceiling or floor effects, and discriminated between ASD and TD groups to varying degrees. Two mentalizing tasks, the TASIT and hinting tasks, demonstrated the largest group differences in performance, with the Benton, BLERT, emotional motion, ER‐40, mind in the eyes, and RAD also producing small to medium effects. These tasks may be useful as baseline or outcome measures for psychosocial interventions [e.g., SCIT, Turner‐Brown, Perry, Dichter, Bodfish, & Penn, 2008; PEERS program; Laugeson, Frankel, Gantman, Dillon, & Mogil, 2012] and pharmacological treatments [e.g., oxytocin trails, see Preti et al., 2014 for review] aimed at targeting social cognition deficits in adults with ASD. Three other tasks (biological motion, CToM, trustworthiness) showed adequate reliability but poor abilities to differentiate groups and may therefore be limited in their use within autistic adult populations. Meanwhile within the TD group, internal reliability was lower on several measures (BLERT, emotional motion, ER‐40, hinting task), and tasks did not align as neatly within a clear factor structure as it did within the ASD group, suggesting that many social cognitive measures used in ASD research may perform differently in non‐clinical samples.

Effect sizes for group differences were largest on two mentalizing/theory of mind tasks, the TASIT and hinting task. The medium to large effects they produced in the current study suggest these tasks require nuanced social inferences, and may be more sensitive measures of mentalizing in intellectually‐able adults with ASD than traditional false belief tasks commonly used with children [Bloom & German, 2000]. Significant effects were also found for several emotion processing (BLERT, ER‐40, and emotional biological motion) and social perception (Benton) measures, indicating that face and emotion processing remain areas of difficulty for adults with ASD. However, because these tasks produced smaller effects (*d* = 0.27–0.49) than the hinting task and the TASIT (*d*s = 0.62 and 0.70, respectively), they are recommended primarily for studies and clinical trials with large enough samples and power to detect small to medium effects.

Social cognition performance across most tasks was strongly related to general cognitive ability and verbal IQ scores, especially for the ASD group. This suggests that individuals with ASD may rely on general cognitive processes to complete social cognitive tasks, with neurocognitive ability in ASD serving as a compensatory factor in social cognitive performance. Results of the factor analysis align with this interpretation, as tasks with stronger correlations with IQ loaded onto the social appraisal factor, suggesting these higher order tasks in particular may require more cognitive resources, with performance driven not just by social cognitive ability but intellectual ability as well. On several tasks (e.g., RAD, Eyes, CToM), IQ correlated highly for both ASD and TD groups, indicating that these tasks in particular may tap neurocognitive skill in addition to social cognitive ability. Future research may seek to determine how specific aspects of neurocognition (e.g., processing speed, working memory, inhibitory control) relate to social cognitive performance in ASD.

Three tasks showed strong reliability, but failed to discriminate the TD and ASD groups (CToM, biological motion, trustworthiness). The CToM is a nonverbal task of metalizing depicting simplistic narratives in comic strip form. This task may be less relevant for revealing social cognitive differences in the intellectually‐able ASD adults assessed here. Similarly, this population did not demonstrate impairments on the biological motion task, which requires categorical differentiation of biologically‐coordinated point light displays from those moving non‐biologically. However, this task does discriminate ASD from TD samples when reaction time or brain activation patterns are examined [Freitag et al., 2008], suggesting that it may perform better when used more sensitively than just examining accuracy. Finally, consistent with the SCOPE study [Pinkham et al., 2015], the trustworthiness task did not discriminate between groups, and was weakly or negatively correlated with performance on other social cognitive tasks. This suggests this task should not be used as an outcome measure in studies examining social cognitive ability in adults with ASD.

As has been suggested in prior work [Sasson et al., 2011], we also found support for a two‐factor structure of social cognition for adults with ASD. Tasks measuring social perception and emotion processing loaded onto the first factor, indicating a relationship in performance between face and affect recognition. Contrary to predictions, the mind in the eyes task and CToM tasks loaded onto the social perception factor rather than the anticipated social appraisal factor. Although both are conceptualized as higher‐order theory of mind tasks, our results suggest these tasks more closely approximate the lower‐order perceptual processing associated with face and affect recognition. The Eyes task may be loading onto this factor because it includes elements consistent with social perception tasks (e.g., selecting emotional states to describe a static facial feature). Indeed, previous work has used this task as an emotion processing measure [Quintana, Guastella, Outhred, Hickie, & Kemp, 2012; Tonks, Williams, Frampton, Yates, & Slater, 2007], Although the Eyes task did demonstrate acceptable psychometric properties, the factor loadings suggest the Eyes task may not be suitable as the primary or exclusive measure of mentalizing/theory of mind in adults with ASD.

As predicted, the TASIT and hinting task aligned with the social appraisal factor in ASD, as did the trustworthiness task, which measures the ability to make a more complex evaluative judgment from faces than categorical emotion recognition. However, in contrast to its designation as a social perception task by the original SCOPE study [Pinkham et al., 2013], the RAD also corresponded to the social appraisal factor. Because the RAD requires participants to generalize understanding of relationships to other scenarios, this measure conceptually aligns more with the higher‐order judgments made in the social appraisal tasks in this study.

While the tasks assessed here generally showed strong utility for assessing social cognition in adults with ASD, results indicated weaker performance in TD adults. Not only was reliability lower, but also the correlations between tasks were weaker. As in the ASD sample, social appraisal tasks, particularly TASIT, showed strong properties; however, the hinting and trustworthiness tasks showed the lowest reliability and validity, suggesting these tasks in particular may not be useful in nonclinical samples or comparison studies. This may be because these measures were designed for use in clinical samples, and as a result, researchers interested in examining social cognition in TD populations may choose to use other tasks developed and designed for nonclinical samples.

The social cognitive performance reported here for ASD shares some similarities with the schizophrenia sample included in the original SCOPE study [Pinkham et al., 2015]. Individuals with ASD and those with schizophrenia both performed worse on all social cognitive tasks relative to TD controls with the exception of the trustworthiness task, with each clinical group demonstrating moderate to strong effect size differences compared to controls [Pinkham et al., 2015; Pinkham et al., 2017]. However, the factor structures of social cognition for ASD and schizophrenia differed, with a two factor structure of social cognition emerging here for ASD but a single factor emerging for schizophrenia [Browne et al., 2016]. Although this distinction may indicate that the underlying structure of social cognition differs between the two conditions, such conclusions are speculative and should be interpreted with caution given that the ASD and schizophrenia samples across studies differed in important ways, including on age, gender, ethnic composition, and level of intellectual functioning. Future studies may remedy this by pursuing a systematic and controlled comparison between the two conditions to determine whether social cognition relates differentially to neurocognition and social functioning in ASD and schizophrenia

The results reported here should be interpreted with several limitations in mind. First, while the tasks assessed in the current study were nominated for evaluation by experts from the fields of both ASD and schizophrenia, the final consensus on which tasks to evaluate as part of SCOPE were made with a focus on clinical trials of schizophrenia. Although ASD and schizophrenia overlap considerably in social cognitive performance [Sasson et al., 2011], and many of the tasks included have been included—even developed (i.e., mind in the eyes)—in autism research studies, task selection may have differed if generated by a consensus panel of social cognitive researchers of autism. Future work is encouraged to evaluate additional social cognitive measures for use with adults with ASD, examine the relative differences between measures within domains to prevent redundancy in construct measurement, and promote standardization across laboratories and clinical trials. This study is also limited by the types of reliability and validity assessed. It is unclear, for instance, whether the tasks assessed here are recommended for longitudinal studies, for younger or less intellectually‐able populations, or for more diverse populations. Future research is encouraged to determine whether performance on these tasks differ for females with ASD and within minority populations. This study also did not assess task test–retest reliability, which is often an important consideration for use in clinical trials in which testing may occur over multiple occasions. Moreover, the criterion prediction validity of these measures remains largely unknown in ASD, and additional studies are needed to assess how social cognition and its subdomains predict functional outcomes. Finally, the results of the factor analysis should be interpreted with caution, as we used a data‐driven technique that may be affected by idiosyncrasies in our sample. In particular, this may have been the case for our TD group, which differed characteristically from the TD group included in the original SCOPE study [Pinkham et al., 2015; Browne et al., 2016] and precludes comparison between factor analyses in the two studies. Because the TD participants were recruited here to match our ASD sample, they tended to be younger, more male, and have higher IQs than the TD participants in the original SCOPE study. These sample differences may also explain differences in task reliability for TD controls in this study compared to SCOPE. Future work should seek to replicate the factor structure and reliability reported here in other populations using confirmatory rather than exploratory analyses.

These limitations notwithstanding, this is the first large‐scale psychometric evaluation of social cognitive tasks for adults with ASD. By selecting measures previously nominated and validated [Pinkham et al., 2015], results from this study provided strong psychometric evidence for many mentalizing, emotion processing, and basic social perception measures. Although all tasks aside from the trustworthiness task performed well, the hinting task and TASIT emerged as the most promising tasks given their strong psychometric properties and large effect size differences between ASD and TD controls. Our results also suggest meaningful distinctions between social perception and social appraisal abilities in adults with ASD, which may have application for treatment and discovering significant predictors of social functioning and outcomes. In sum, this psychometric evaluation of social cognitive tasks for adults with ASD serves as a first step toward creating a gold standard battery that can identify relevant domains of social cognition in ASD, assess its relevance to functional outcomes, and facilitate future research and clinical trials.

## Conflict of Interest

The authors have no conflicts of interest.
